# Transcriptional cooperation of PBX1 and PAX6 in adult neural progenitor cells

**DOI:** 10.1038/s41598-021-99968-5

**Published:** 2021-10-25

**Authors:** Ann-Christin Hau, Elise Mommaerts, Vera Laub, Tamara Müller, Gunnar Dittmar, Dorothea Schulte

**Affiliations:** 1 Neurological Institute, Edinger Institute, University Hospital, Goethe University, Heinrich-Hoffmann-Str. 7, 60528 Frankfurt, Germany; 2 NorLux Neuro-Oncology Laboratory, Department of Oncology, Luxembourg Institute of Health, 84, Val Fleuri, 1526 Strassen, Luxembourg; 3 National Center of Pathology, Laboratoire National de Santé, 1 rue Louis Rech, 3555 Dudelange, Luxembourg; 4 Quantitative Biology Unit, LUXGEN, Luxembourg Institute of Health, 1 A-B Rue Thomas Edison, 1445 Strassen, Luxembourg

**Keywords:** Neural stem cells, Gene regulation

## Abstract

PAX6 is a highly conserved transcription factor and key regulator of several neurogenic processes, including the continuous generation of dopaminergic/GABAergic interneurons in the adult ventricular-subventricular (V-SVZ) neurogenic system in mice. Here we report that PAX6 cooperates with the TALE-homeodomain transcription factor PBX1 in this context. Chromatin-immunoprecipitation showed that PBX1 and PAX6 co-occupy shared genomic binding sites in adult V-SVZ stem- and progenitor cell cultures and mouse embryonic stem cells, while depletion of *Pbx1* revealed that association of PAX6 with these sites requires the presence of PBX1. Expression profiling together with viral overexpression or knockdown of *Pax*6 or *Pbx*1 identified novel PBX1-PAX6 co-regulated genes, including several transcription factors. Computational modeling of genome wide expression identified novel cross-regulatory networks among these very transcription factors. Taken together, the results presented here highlight the intimate link that exists between PAX6 and TALE-HD family proteins and contribute novel insights into how the orchestrated activity of transcription factors shapes adult V-SVZ neurogenesis.

## Introduction

**Figure 1:**
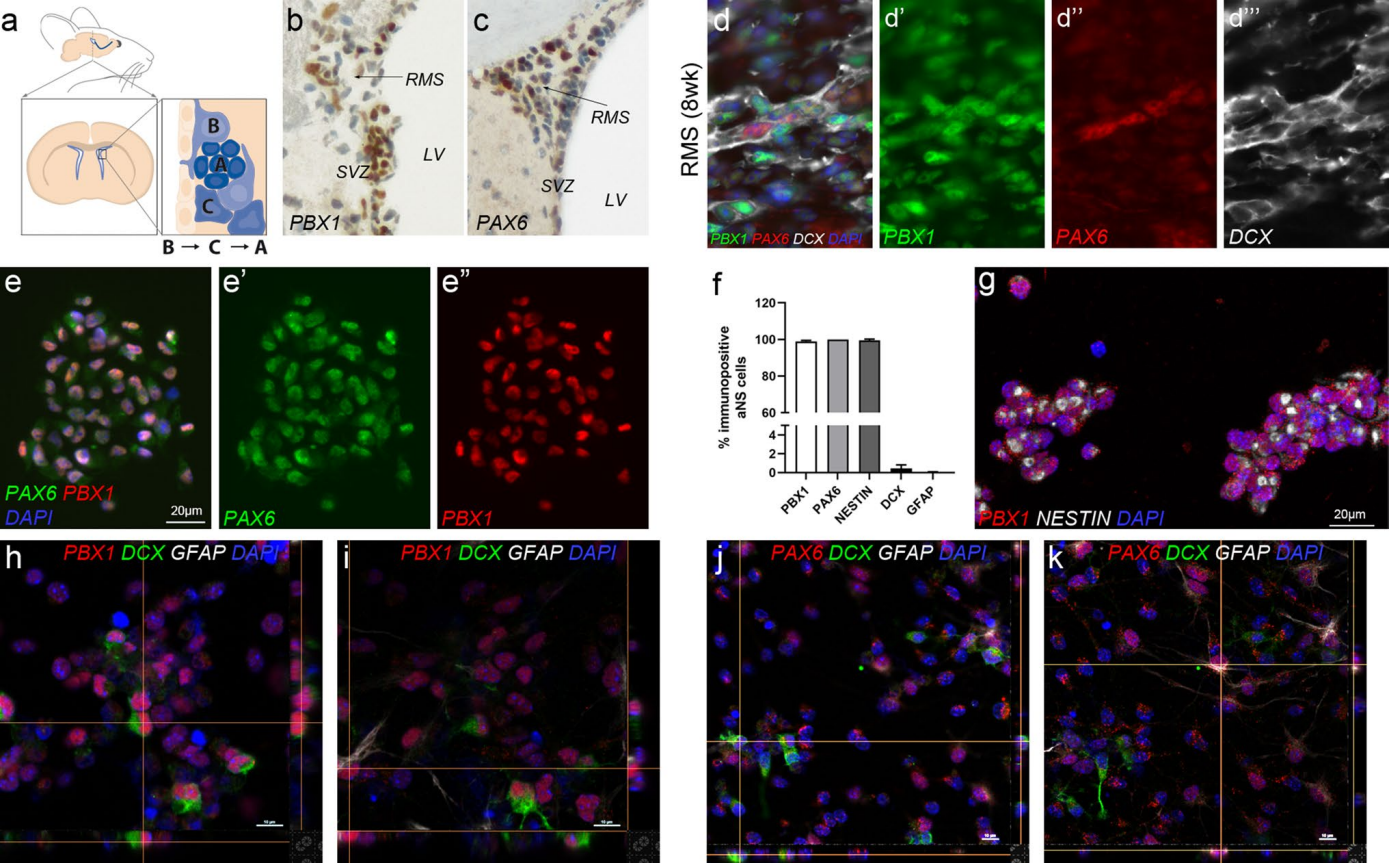
Characterization of PBX1- and PAX6-immunoreactive cells in vivo and in vitro. (**a**) Schematic representation of the V-SVZ. (**b**,**c**) Presence of PBX1 and PAX6 (brown) in the adult mouse SVZ including parts of the RMS. (**d**–**d’’’**) Immunohistochemical staining for PAX6, PBX1, and DCX in the caudal, i.e. V-SVZ proximal, RMS. (**e**) Full view of a single aNS stained for PBX1 and PAX6; the two transcription factors co-localize to cell nuclei. (**f**) Proportion of PBX1+, PAX6+, NESTIN+, DCX+ or GFAP+ cells in sphere-forming aNS (*n* = 4–5; 2150 cells on average counted per experiment; error bars indicate S.D.). (**g**) Representative image of aNS stained for PBX1 and NESTIN; confocal image series, maximum intensity projection. (**h**,**i**) Spheres differentiated for 24 h and stained for PBX1, DCX, and GFAP; single confocal images at two different Z-positions are shown, focusing on PBX1/DCX + cells (**h**) or PBX1/GFAP + cells (**i**); Z-projections are shown on the side of each image. (**j**,**k**) 24 h differentiated cells stained for PAX6, DCX, and GFAP; single confocal images at two different Z-positions, focusing on PAX6/DCX + cells (**j**) or PAX6/GFAP + cells (**k**); Z-projections shown on the side. See also Fig. S1.

Cellular plasticity in the adult mammalian brain occurs through the generation of new neurons in selected germinal niches. In rodents, these are the subgranular zone in the dentate gyrus of the hippocampus, the ventricular-subventricular zone (V-SVZ) in the lateral walls of the lateral ventricle, and a more recently recognized stem cell niche in the hypothalamus^1–3^. Neurogenesis in all stem cell niches starts with the activation of quiescent, non-proliferative stem cells and progresses over an intermediate population of transient amplifying progenitors (TAPs) to young neurons, termed neuroblasts (Fig. 1a). Neuroblasts born in the adult V-SVZ migrate in chains along the rostral migratory stream (RMS) into the olfactory bulb (OB), where most of them differentiate into GABAergic interneurons and integrate into existing neuronal circuits^1^. Many transcription factors promote neuronal differentiation and/or control lineage specification of adult born progenitor cells of the V-SVZ, including PAX6^4,5^, DLX1/DLX2 and DLX5/DLX6^6,7^, MEIS2^8,9^, PBX1^10^, SP8^11^, TBR2^12^, VAX1^13^, tailless/TLX^14^, and SOX4/SOX11^15^. However, if and to what extent these proteins cooperate in the regulation of cell type-specific genetic programs is still poorly defined.

The three amino acid loop extension (TALE) homeodomain (HD) transcription factor PBX homeobox 1 (Pre-B cell leukemia transcription factor 1, *Pbx1*) is expressed in TAPs and neuroblasts of the V-SVZ and some types of adult generated OB interneurons^10^. Targeted deletion of *Pbx1* in different cell populations of the V-SVZ stem cell niche elicits distinct outcomes. Deletion in TAPs directs them from a neurogenic to an oligodendrogliogenic fate, deletion in neuroblasts results in cell death, and deletion in differentiating dopaminergic neurons disturbs their maturation and the expression of characteristic dopamine pathway genes^10,16^. *Pbx1* thus plays pivotal roles in the generation, survival, and terminal differentiation of neurons in the adult V-SVZ. Mechanistically, PBX1 cooperates with another TALE-HD protein, MEIS2, to control gene expression. Among the genes jointly regulated by MEIS2 and PBX1 is doublecortin (*Dcx*), coding for a microtubule-associated protein, which is expressed by all newborn young neurons in the embryonic and adult CNS^8,10,17^. Binding of PBX1 and MEIS2 to a *Dcx* proximal enhancer follows a strict temporal sequence, whereby PBX1 associates with its recognition site already at a time when the *Dcx* gene locus is compacted by histone H1 and hence significantly prior to *Dcx* transcriptional activation. As soon as cells start to differentiate, MEIS2 enters the cell nucleus, joins PBX1 at the DNA and recruits the chromatin-modifying enzyme Poly(ADP-ribose)-polymerase 1 (PARP1) into the complex^9,18,19^. This then leads to local chromatin decompaction and facilitates the binding of additional regulatory proteins, such as histone modifying enzymes, chromatin remodeling complexes or other transcription factors^18,20–22^. It remains unanswered, however, whether these events are executed by the PBX1-MEIS2 dyad alone or whether additional transcription factors also participate. A good candidate is the paired-type homeodomain protein PAX6. PAX6 is a key neurogenic fate determinant in the embryonic telencephalon and adult V-SVZ neurogenic niche^4,23–25^. PAX6 can induce the acquisition of a GABAergic, dopaminergic neuronal phenotype in adult V-SVZ neural progenitors but fails to do so when *Meis2* is knocked down, implicating MEIS2 as cofactor of PAX6 in this neurogenic niche^8^. PAX6 also co-precipitates with PBX1, raising the possibility that both proteins functionally interact^8,10^. Experimental evidence for this, however, has been missing. Here, we used chromatin-immunoprecipitation (ChIP) and gene expression profiling to experimentally test the hypothesis that PBX1 and PAX6 cooperate in common gene regulation of adult V-SVZ progenitor cells.

## Results

### PBX1 and PAX6 bind to adjacent sites in the regulatory regions of shared target genes

Expression of *Pbx1* and *Pax6* has been reported in TAPs in the V-SVZ and in migrating neuroblasts within the V-SVZ, RMS and OB^4,10^. We confirmed these reports by immunohistochemical staining of paraffin-embedded sections of the adult mouse V-SVZ (Fig. 1b,c). Co-immunofluorescent staining of PBX1, PAX6 and DCX on cryosections of the caudal RMS close to the V-SVZ further demonstrated widespread presence of PBX1 in DCX+ neuroblasts, some of which co-labeled for PAX6 (Fig. 1d). These findings agree with previous results. An earlier quantification of the proportion of PBX1+ cells in the adult murine forebrain found that approximately 82% of TuJ1 (neuronal βIII-tubulin (Tubb3))-positive cells in the RMS are immunoreactive for PBX1^25^. *Pax6* expression in the RMS, in turn, follows a distinct spatial–temporal dynamic with low numbers of PAX6-immunoreactive cells at the beginning of the RMS close to the V-SVZ and increasing numbers of PAX6+ neuroblasts towards the central RMS^4^. Stem- and progenitor cells taken from the adult mouse V-SVZ can be propagated in vitro in the presence of epidermal growth factor (EGF) and basic fibroblast growth factor (FGF2) as sphere-forming cellular aggregates, termed adult neurospheres (aNS), and will differentiate into neuroblasts, astro- and oligodendroglia under appropriate culture conditions. PBX1 and PAX6 exhibited strong immunoreactivity in virtually all aNS cells (Fig. 1e). PBX1 hereby was exclusively nuclear while PAX6 showed strong nuclear as well as weaker cytoplasmic staining (Fig. 1e). The vast majority of sphere-forming cells were neural stem- and progenitor cells, as demonstrated by fluorescence-activated cell sorting (FACS) of aNS for integrin α6 (ITGA6; Fig. S1a,b). In support of this, triple-immunostaining for PBX1 or PAX6 together with NESTIN, an intermediate filament also specific for neural progenitor cells, further revealed that virtually all aNS cells were immunoreactive for NESTIN, as well as for PBX1 and PAX6, respectively (Fig. 1f,g, Fig. S1e). By contrast, only very few cells adopted a neuronal or glial cell fate under these conditions, recognizable by the low numbers of cells immunoreactive for DCX or the neuronal epitope PSA-NCAM and even fewer GFAP-expressing astrocytes in these cultures (Fig. 1f, Fig. S1c–e). When taken in culture, adult V-SVZ aNS thus consist of undifferentiated, sphere-forming cells, all of which co-express PBX1 and PAX6. Exchanging EGF and FGF2 for BDNF and plating aNS on laminin induced rapid cellular differentiation, with an increase of DCX-expressing neurons to 21.49 (± 4.89) %, of GFAP+ astrocytes to 18.73 (± 1.46) %, and a decrease of undifferentiated, NESTIN+ cells to 1.5 (± 0.97) % within 24 h (Fig. S1f.). Virtually all DCX+ or GFAP+ cells thereby co-labeled for PBX1 and PAX6 (Fig. 1h–k, Fig. S1f.). The proportion of *Pbx1*/*Pax6* co-expressing neuroblasts in vitro thus exceeded the percentage of co-expressing cells among migrating neuroblasts in vivo, at least when DCX+ cells after 24 h of in vitro differentiation were compared to DCX+ cells in the caudal RMS adjacent to the V-SVZ.

**Figure 2:**
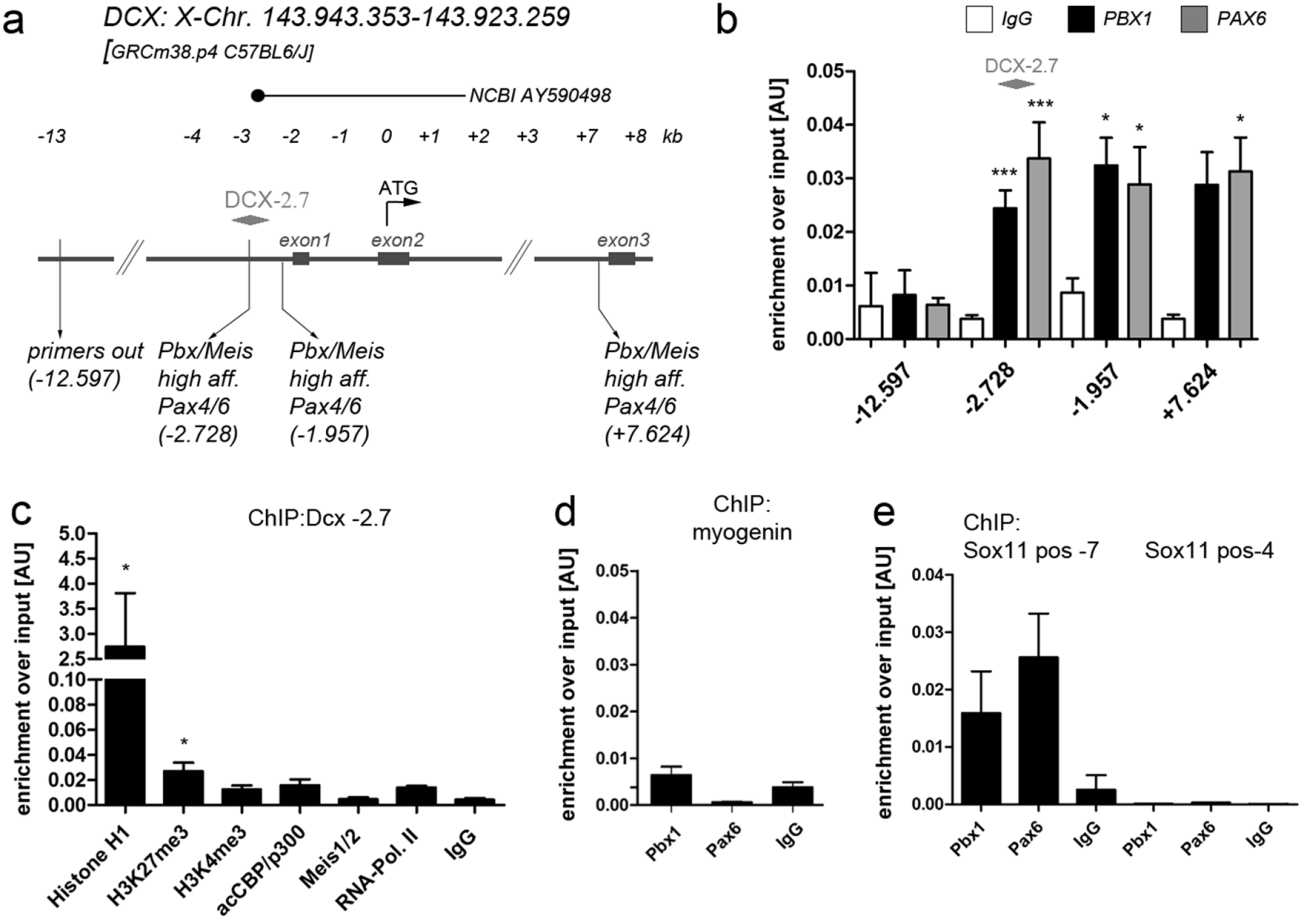
Chromatin changes and PBX1-PAX6 co-occupancy at regulatory regions of *Dcx* and *Sox11*. (**a**) Schematic drawing of the *Dcx* genomic locus analyzed by ChIP. (**b**) ChIP-qPCR in aNS for PBX1, PAX6 und non-specific control IgGs at the *Dcx* locus. (**c**) ChIP-qPCR for *Dcx* in aNS with the antibodies indicated. (**d**) PBX1-ChIP at the myogenin promoter. (**e**) ChIP-qPCR for PAX6 and PBX1 at the Sox11 -7 site of ^28^. In all figures, statistical significance of ChIP-qPCR with the antibodies used for ChIP over isotype-specific IgGs is given as *: *p* < 0.05, **: *p* < 0.01, ***: *p* < 0.001. Error bars represent S.E.M..

Because conditional ablation of either *Pbx1* or *Pax6* in adult V-SVZ stem- and progenitor cells impairs adult neurogenesis, we examined whether PBX1/PAX6 associate with known regulatory regions of the neuron-specific gene *Dcx*^4,10^. A high affinity binding site for PBX and MEIS proteins, flanked by a putative PAX6 binding site, is located 2728 base pairs (bp) upstream of the *Dcx* start codon (termed *Dcx-2.7*) within a characterized proximal enhancer of the *Dcx* gene (Fig. 2a)^26,27^. In V-SVZ-derived young neurons and hence in cells in which PBX1, MEIS2 and PAX6 are present in the cell nucleus, this position is bound by all three transcription factors^8,10,18^. In neural stem- and progenitor cells in the adult SVZ in vivo and in aNS in vitro, however, PBX1 and PAX6 are nuclear, whereas MEIS2 is not (Fig. 1e;^9,19^). We therefore examined whether PBX1 and PAX6 occupy the *Dcx-2.7* binding site in aNS. Chromatin-immunoprecipitation followed by quantitative PCR (ChIP-qPCR) for PBX1 or PAX6 successfully enriched both transcription factors at the *Dcx-2.7* site, while validated IgG control antibodies were unsuccessful (Fig. 2b, position -2728). Inspection of the wider genomic sequence surrounding the transcriptional start site of the *Dcx* gene identified additional sequence motifs consisting of a MEIS-PBX consensus binding site close to a putative PAX6 binding motif at 1957 bp upstream of the *Dcx* start codon, i.e. within the *Dcx* promoter/proximal enhancer, and 7624 bp downstream of the start codon, which corresponds to the intron located between exons 2 and 3 (Fig. 2a). PBX1 and PAX6 also bound to the chromatin fiber at these positions (Fig. 2b). By contrast, a site 12,597 bp upstream of the Dcx start codon (‘primers out’) was not enriched by the PBX1- or PAX6-specific antibodies (Fig. 2b).

The *Dcx-2.7* site was compacted by the linker histone H1.4 and weakly positive for the repressive histone modification H3K27^me3^ but not for the mark of transcriptionally active promoters H3K4^me3^, establishing that PAX6 and PBX1 can bind to predominantly inactive chromatin (Fig. 2c). In agreement with this, we also could not detect the active form of the histone acetyl transferase CBP/p300 (acCBP/p300), RNA polymerase II (RNApolII), or MEIS1/2 at *Dcx-2.7* in aNS (Fig. 2c). PBX1 or PAX6 did not associate with a known PBX1 binding site in the muscle specific gene *myogenin* (*Myog*) in aNS, demonstrating that both transcription factors do not bind to the regulatory region of a lineage-inappropriate gene (Fig. 2d). PAX6 cooperates with the Brg1/BAF ATP-dependent chromatin remodeling complex to drive expression of a set of cross-regulating transcription factors that control neuronal fate acquisition in the adult SVZ neurogenic niche^28^. One of these transcription factors is *Sox11*. Although we could not confirm binding of PAX6 to a position 4 kb upstream of the Sox11 transcriptional start site (TSS; position -4 in^28^), we observed strong binding of both, PAX6 and PBX1, to a site 7 kb upstream of the Sox11 TSS (position -7 in^28^; Fig. 2e). *Dcx* and *Sox11* thus emerge as direct target genes of PAX6 and PBX1 in adult-generated V-SVZ progenitor cells.

### PAX6 fails to associate with selected genomic bindings sites in the absence of PBX1

**Figure 3:**
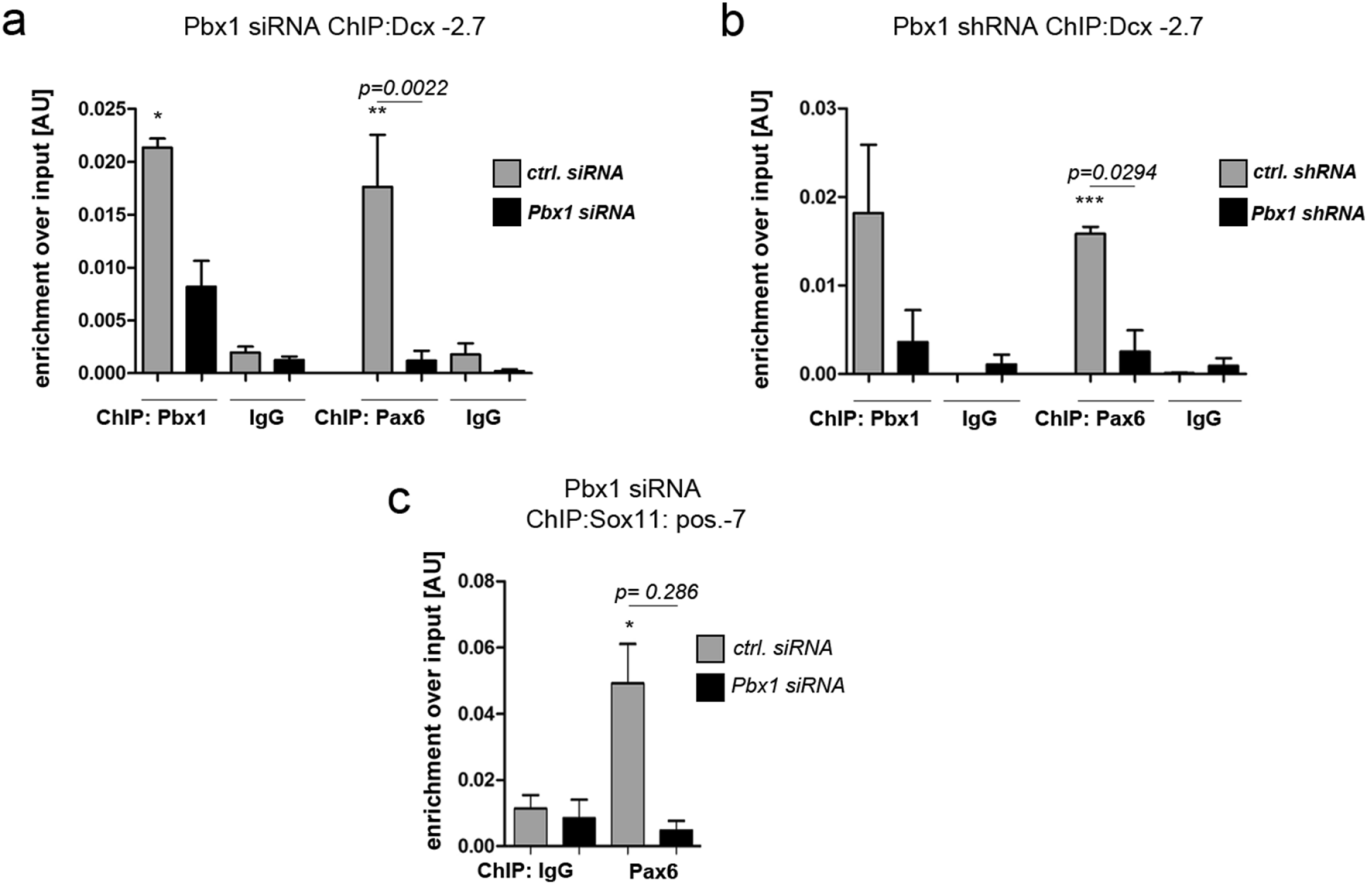
PAX6 fails to associate with the *Dcx*-2.7 and *Sox11(-7)* sites following depletion of *Pbx1*. (**a**, **b**) ChIP-qPCR for PAX6 at the *Dcx-2.7* site in aNS following KD of *Pbx1* by siRNAs (**a**) or following lentiviral delivery of shRNAs (**b**). (**c**) ChIP-qPCR for PAX6 at the Sox11(-7) site in aNS following KD of *Pbx1*. Statistical significance of ChIP-qPCR with the antibodies specific for PBX1 or PAX6, respectively, over isotype-specific IgGs is given as *: *p* < 0.05, **: *p* < 0.01, ***: *p* < 0.001 (indicating enrichment relative to IgG). Statistical significance between ChIP results obtained with *Pbx1*KD and ctrl samples (black and grey bars, respectively) is given as *p* = numerical value (indicating the effect of *Pbx1*KD). Error bars represent S.E.M. See also Fig. S2.

To test whether PAX6 requires PBX1 for binding to these positions in the *Dcx* and *Sox11* regulatory regions, aNS isolated from the adult V-SVZ were transfected with siRNA against *Pbx1* before they were processed for ChIP-qPCR (Fig. S2a). As expected from a successful knockdown (KD) of *Pbx1*, binding of PBX1 to the *Dcx-2.7* site was significantly reduced (Fig. 3a). Notably, binding of PAX6 at this position was also largely diminished, suggesting that association of PAX6 with this genomic site depends on the presence of PBX1 (Fig. 3a). Depletion of PBX1 by lentiviral transduction of shRNAs also diminished binding of PBX1 and PAX6 to the *Dcx-2.7* site (Fig. 3b; Fig. S2b,c). In addition, we observed a marked reduction of PAX6 binding to the *Sox11* pos-7 site (Fig. 3c). PAX6 protein in aNS was unaltered following *Pbx1* KD, making it highly unlikely that the diminished PAX6 binding to these two genomic sites resulted from a general loss of PAX6 protein in the cells (Fig. S2d). Collectively, these results argue that PAX6 requires PBX1 for association with the regulatory regions of two of its target genes in undifferentiated adult SVZ-derived progenitor cells.

**Figure 4:**
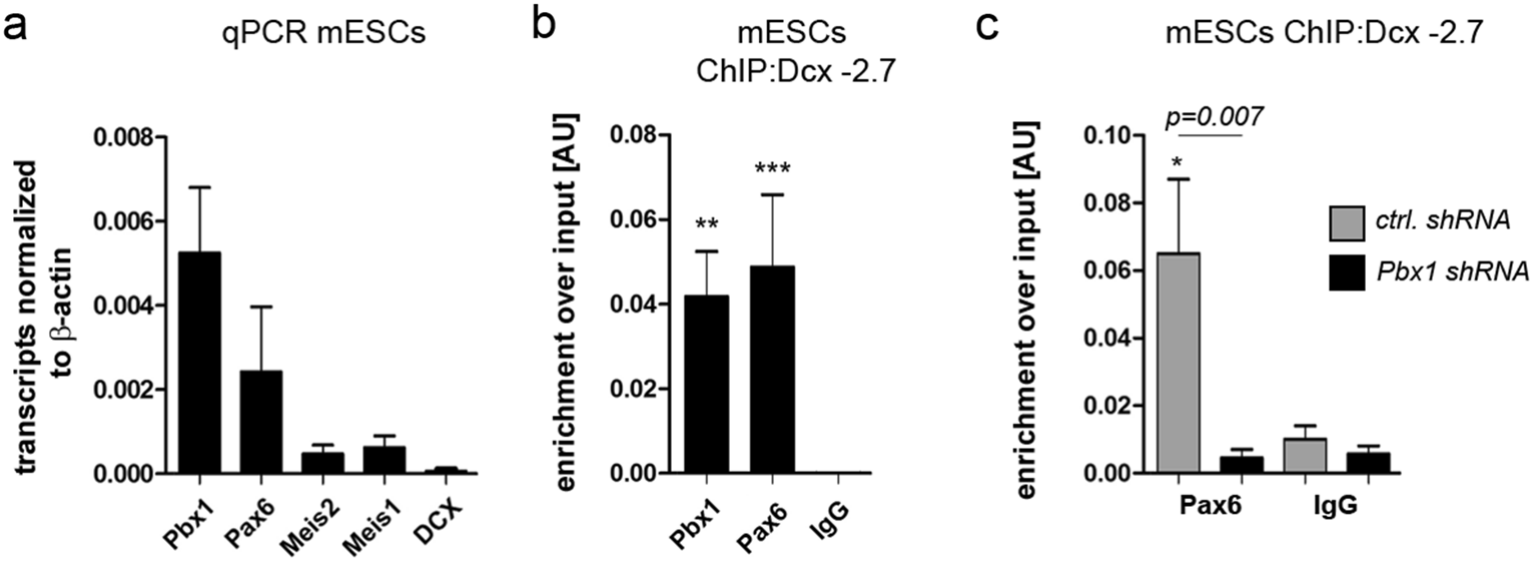
PBX1 recruits PAX6 to *Dcx*-2.7 and *Sox11(-7)* in murine embryonic stem cells. (**a**) Transcript expression of the genes indicated in mESCs. (**b**) ChIP-qPCR for PBX1 and PAX6 at *Dcx*-2.7 in ESCs. (**c**) PAX6 does not associate with *Dcx*-2.7 upon *Pbx1* KD. In all figures, statistical significance of ChIP-qPCR with the PBX1 or PAX6 antibodies over isotype-specific IgGs is given as *: *p* < 0.05, **: *p* < 0.01, ***: *p* < 0.001. Statistical significance between ChIP results obtained with *Pbx1*KD and ctrl samples (black and grey bars, respectively) is given as *p* = numerical value. Error bars represent S.E.M.

To extend these findings, we asked whether PBX1 might already be present at the *Dcx* promoter/enhancer in embryonic stem cells (ESCs) and whether it also facilitates PAX6-chromatin binding in this setting. E14Tg2a.IV murine Sox1-GFP knock-in ESCs (46C) grown in LIF-containing medium without feeder cells^29^ showed robust expression of *Pbx1* and *Pax6*, but not of *Meis1* or *Meis2* (Fig. 4a). Consistent with the undifferentiated nature of the cells, they were also negative for *Dcx* (Fig. 4a). As we had observed in the aNS cultures from the adult mouse V-SVZ, PBX1 and PAX6 associated with the shared *Dcx-2.7* genomic binding site prior to *Dcx* expression (Fig. 4b) and *Pbx1* KD reduced PAX6 binding to this site (Fig. 4c). PBX1, hence, facilitates PAX6 binding to DNA also in this biological setting.

### Shared and unique gene expression changes following *Pbx1* and *Pax6* manipulation in V-SVZ neurospheres

**Figure 5:**
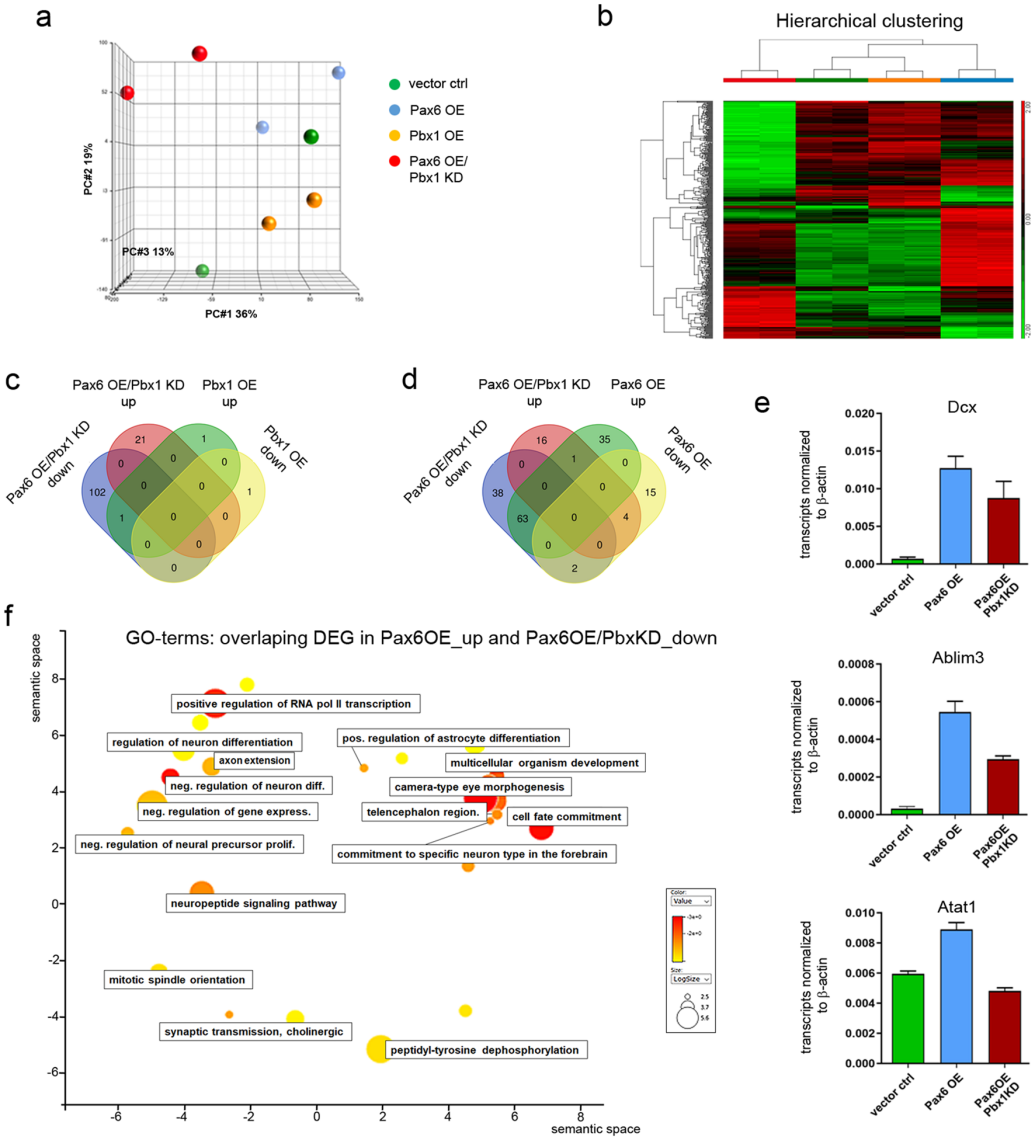
Identification of PAX6-PBX1 co-regulated genes by Affymetrix Mouse Gene 1.0 ST arrays profiling. (**a**) PCA plot of transcriptomes from adult V-SVZ-neurospheres differentiated under standard conditions (vector ctrl-green), neuronally differentiated (*Pax6*OE-blue) and upon *Pbx1* manipulation (*Pbx1*OE-yellow or *Pax6*OE/*Pbx1*KD-red) profiled by Affymetrix GeneChip microarray. Each dot represents an individual array/sample (N = 2 samples per condition). (**b**) Heatmap of differentially expressed genes (DEGs) between all dataset conditions (as determined by ANOVA), comprising in total 382 DEGs with FDR ≤ 0.05 and no cut-off in fold change (FC). Heatmap was computed using Partek Genomics Suite version 7 (https://www.partek.com/partek-genomics-suite/). (**c**) Venn Diagram comparing DEGs (FDR ≤ 0.05, − 1.5 ≤ FC ≥ 1.5) up- and/or downregulated upon *Pax6*OE/*Pbx1*KD or *Pbx1*OE, respectively. (**d**) Venn Diagram comparing DEGs (FDR ≤ 0.05, − 1.5 ≤ FC ≥ 1.5) up- and/or downregulated upon *Pax6*OE/*Pbx1*KD or *Pax6*OE, respectively. (**e**) Relative transcript levels of exemplary PBX1-PAX6 co-regulated genes, e.g. *Dcx, Ablim3 and Atat1* in V-SVZ-neurospheres differentiated under standard conditions (vector ctrl-green), neuronally differentiated (*Pax6*OE-blue) and upon *Pbx1* manipulation (*Pax6*OE/*Pbx1*KD-red) validated by qPCR. (**f**) Graphical summary of GO terms matching `Biological Processes` statistically over-represented in the list of PBX1-PAX6 co-regulated genes (REVIGO). Semantic modalities sharing common ancestors in the GO database are closer together, representing clusters characterizing highly related biological annotations. See also Fig. S3.

To test whether the observed interaction of PAX6 and PBX1 plays out on a more general scale, we performed genome-wide expression analyses. We reasoned that when PBX1 was indeed needed for PAX6’ neurogenic activity in the adult V-SVZ stem cell niche, genes that become transcriptionally upregulated following *Pax6* viral OE in adult stem- and progenitor cells may fail to do so when *Pax6* transduction was combined with *Pbx1* KD. We took advantage of our previous finding that a differentiation period of 10 h is sufficient to elicit gene expression changes that can be detected by Affymetrix gene expression profiling^18^. Primary aNS were subjected to one of the following four treatments (1) infection with pCLIG, GFP-expressing retroviral vector viruses (vector ctrl)^30^, (2) infection with pCLIG expressing *Pbx1* (transcript variant 1b; *Pbx1*OE), (3) transfection with non-targeting siRNAs followed by infection with pCLIG expressing *Pax6* (*Pax6*OE), and (iv) transfection with siRNAs targeting *Pbx1* followed by infection with pCLIG expressing *Pax6* (*Pax6*OE/*Pbx1*KD). 48 h later, the cells were differentiated for 10 h on laminin-coated dishes by removal of EGF and FGF2 from the culture medium before they were processed for Affymetrix GeneChip hybridization. Principal component analysis captured 68% of variance within the analyzed dataset (PC1 36%, PC2 19% and PC3 13%) comprising *Pbx1*OE and/or *Pax6*OE/*Pbx1*KD as well as respective controls (n = 2) and revealed 382 genes differentially expressed within the dataset (FDR ≤ 0.05). Replicate samples predominantly clustered together except vector ctrl (Fig. 5a, green). Surprisingly, whereas *Pax6*OE (bright blue in Fig. 5a,b) markedly altered the gene expression compared to vector-transduced cells (green in Fig. 5a,b), *Pbx1*OE had little effect (yellow in Fig. 5a,b). Microarray analysis of differentially expressed genes (DEGs) as determined with combined FDR and an absolute fold change of FC ≥ 1.5 identified 124 genes differentially expressed following *Pax6*OE/*Pbx1*KD (Table S1), but only 3 genes were differentially expressed upon *Pbx1*OE (Table S2), relative to respective controls. In fact, the only gene whose transcript levels were sensitive to *Pbx1*OE and *Pax*6OE/*Pbx1* KD, compared to the respective controls, was *Pbx1* itself. Upon *Pax6*OE, however, 120 DEGs were identified, of which 99 genes showed up- and 21 genes downregulation compared to cells transduced with the empty vector (Table S3). Upregulated genes were primarily associated with Gene Ontology (GO)-terms related to ‘nervous system development’, ‘cell differentiation’, and ‘cell fate commitment’ when analyzed with the DAVID functional annotation tool (Table S4). Elevated expression of *Pax6*, but not *Pbx1*, prior to differentiation hence induced widespread gene expression changes that are consistent with the neuronal differentiation of forebrain neural stem- and progenitor cells. Given that previous work had shown that genetic deletion of *Pbx1* impairs V-SVZ neurogenesis in vitro and in vivo, *Pbx1* seems to be necessary but not sufficient for the production of neurons in this adult germinal niche^10^.

We therefore asked whether *Pbx1* was required for the gene expression changes that *Pax6*OE elicits. Affymetrix GeneChip expression profiling revealed clear differences between the *Pax6*OE/*Pbx1*KD and *Pax6*OE samples (Fig. 5a–d). Relative to *Pax6*OE, transcript levels of 103 genes were significantly reduced and those of 21 genes significantly increased when *Pax6*OE was combined with *Pbx1*KD (Table S1). To illustrate the intersection of genes potentially co-regulated by PBX1 and PAX6, transcripts that are differentially regulated during *Pax6*-directed neurogenesis (*Pax6*OE) and *Pbx1*-*Pax6*-co-dependent neuronal differentiation (*Pax6*OE/*Pbx1*KD) were intersected with the differentially regulated genes identified after *Pbx1*OE (Fig. 5c) or *Pax6*OE (Fig. 5d) and plotted as Venn diagrams. In total, 174 genes were found to be differentially expressed (up or down) in the two datasets. Of these, 70 genes (40% of all DEGs) were altered in both gene sets, indicating joint regulation by PAX6 and PBX1. Notably, most upregulated genes in the *Pax6*OE dataset (63%) were significantly downregulated when *Pax6*OE was combined with *Pbx1*KD (63/99, Fig. 5d, intersection of *Pax6*OE_Up *Pax6*OE/*Pbx1*KD_Down; Table S5). To interrogate their involvement in biological processes, these 63 genes were subjected to the online resource DAVID; GO terms and corresponding p-values were extracted and submitted to REVIGO, a computational approach to reduce functional redundancies in GO term lists and graphically display results^31^. PBX1-PAX6 co-dependent DEGs classified to GO terms such as 'negative regulation of neural precursor cell proliferation', 'regulation of neuron differentiation', and ‘commitment of neuronal cell to specific neuron type in the forebrain' (Fig. 5f), corroborating the notion that PBX1 and PAX6 are synergistically involved in V-SVZ neurogenesis and OB neuronal fate specification and differentiation.

These global gene expression changes were well recapitulated when *Dcx* is taken as proxy for neuron-specific transcript: *Pax6*OE increased *Dcx* expression approximately eightfold, which was diminished by simultaneous KD of *Pbx1* (Fig. 5e). REVIGO analysis also identified additional genes that had not yet been linked to V-SVZ neurogenesis but showed similar regulation profiles to *Dcx*, such as actin binding LIM protein family, member 3 (*Ablim3*) and alpha tubulin acetyltransferase 1 (*Atat1*) (Fig. 5e; Table S3). Notably, these transcripts also exhibit prominent expression within the SVZ-OB neurogenic system (Fig. S3).

### Computational modeling the Affymetrix gene expression results reveals candidate transcription factors for V-SVZ regulatory networks

We next examined the gene expression data for three conditions, *Pax6* OE*, Pax6* OE/*Pbx1*KD and empty vector control, with the web-based analysis tool ISMARA. ISMARA is a computational approach to infer gene regulatory circuitries from high-throughput gene expression data^32^. It models comparative gene expression data and predicts genome-wide regulatory interactions together with the transcription factors that likely drive the observed gene expression changes. Based on a precalculated genome-wide annotation of promoters, ISMARA defines the importance of regulatory motifs for explaining expression differences among samples as ‘motif activity’ and sorts each motif activity by a Z-score.

**Figure 6:**
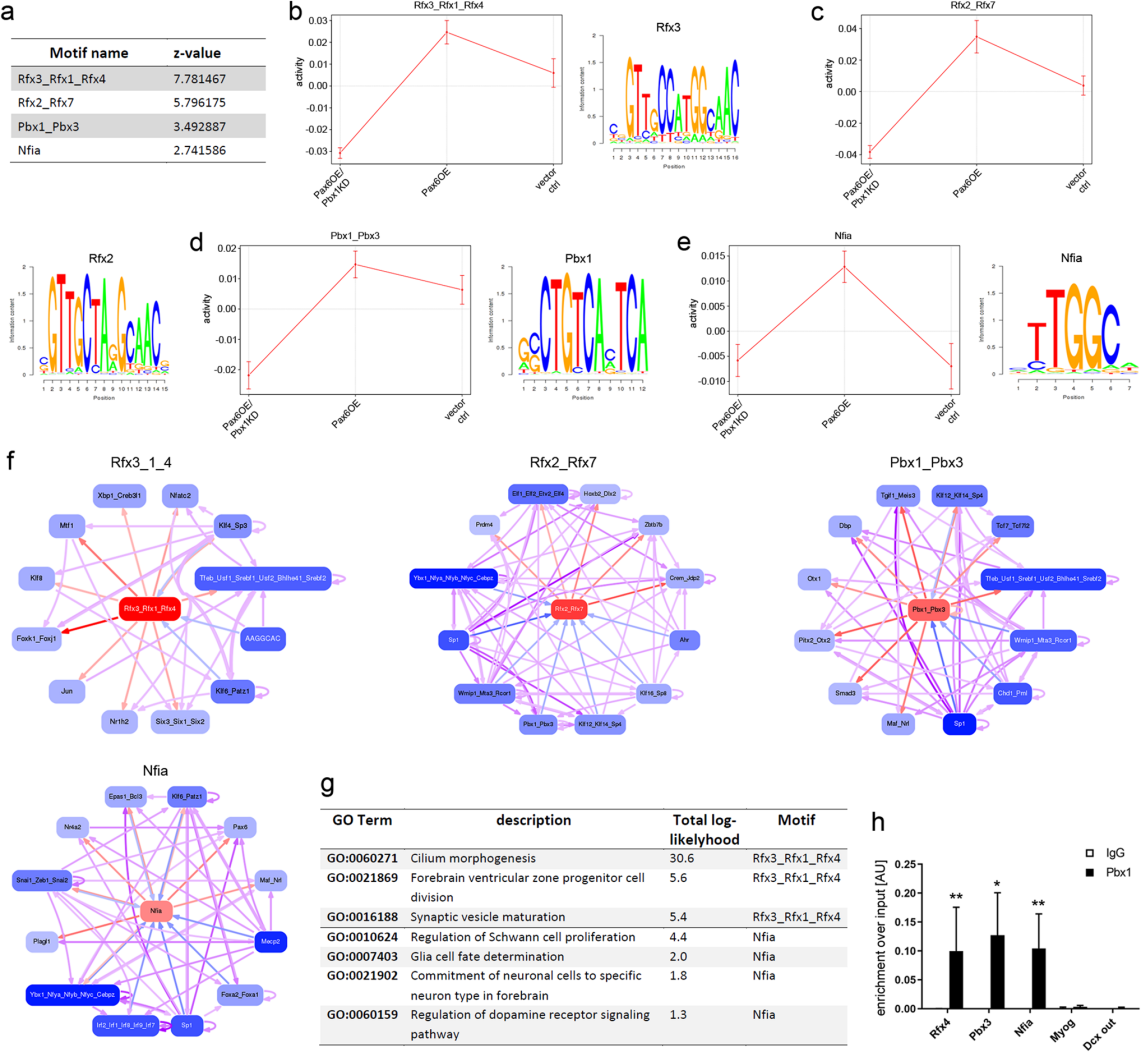
Prediction of transcriptional networks downstream of PAX6-PBX1 by computational modeling. (**a**) Z-scores of four novel transcription factor binding motifs identified by ISMARA analysis to be relevant for the expression differences between the *Pax6*OE, *Pax6*OE/*Pbx1*KD and vector ctrl datasets. (**b–e**) Motif activity score profiles and consensus recognition sequences of the four motifs. The predicted activity of each motif increases upon *Pax6*OE and drops after *Pbx1*KD, relative to empty vector control. (**f**) Graphical representation of regulatory networks centered around the four motifs. Each motif is predicted to directly control the expression of several additional transcription factors, many of which are part of multiple shared networks (extracted from ISMARA online tool; https://ismara.unibas.ch/mara/). (**g**) GO terms associated with the *Rfx3*_*Rfx1*_*Rfx4* or *Nfia* motifs. (**h**) ChIP-qPCR with antibodies for PBX1 or mouse control IgGs confirms prominent PBX1 binding to regulatory regions of *Rfx4*, *Pbx1* and *Nfia*; PBX1-ChIP for the myogenin promoter and the *Dcx*-12.5 site (‘out’) served as additional controls. See also Fig. S4.

Applying this approach to our data identified several key motifs that were associated with differentially expressed genes between conditions. These motifs included consensus binding sequences for known regulators of neurogenesis like *Nfix* (Z-value 2.82;^33,34^), *Sox2* (Z-value 2.09;^35^), *Klf16_SP8* (Z-value 1.62;^36–38^), and *Nfic_Nfib* (Z-value 1.38;^28,39^), as well as recognition motifs of transcription factors that had not yet been implicated in V-SVZ neurogenesis (Table S6). These included *Rfx3_Rfx1_Rfx4* and *Rfx2_Rfx7*, motifs bound by regulatory factor X (Rfx) transcription factors, *Pbx1*_*Pbx3*, bound by PBX1 itself and PBX3, as well as the recognition motif for the nuclear factor I A *Nfia* (Fig. 6a). Because members of each of these groups are expressed in the adult V-SVZ (Fig. S4), we considered these motifs as possible novel regulons of adult V-SVZ neurogenesis. Interestingly, the activity profiles for each motif exhibited a clear increase among transcripts obtained from the *Pax6OE* samples compared to the empty vector control samples and a marked decrease when *Pax6OE* was combined with *Pbx1KD* (Fig. 6b–e). This suggests that transcription factors associated with these motifs are positively linked to *Pax6*-induced neurogenic differentiation and co-regulated by PAX6 and PBX1. We also predicted the target promoters that are likely controlled by these PAX6-PBX1 co-dependent motifs and reconstructed the network of known interactions between them. All four motifs targeted multiple other transcription factors and extensive cross-regulatory networks appeared to exist among them (Fig. 6f). Notably, these networks included several established regulators of V-SVZ neurogenesis, with *Dlx2*^6^ and *Sp8*^38^ targeted by *Rfx2*_*Rfx7*, and *Pax6*^4^ and the key transcription factor of dopaminergic differentiation *Nr4a2/Nurr1* targeted by Nfia as prominent examples. In addition, genes carrying any of these four motifs in their promoters were themselves associated with GO terms related to adult V-SVZ neurogenesis, as exemplarily shown for the *Rfx3_Rfx1_Rfx4* and *Nfia* target families (Fig. 6g; Tables S7, 8). Importantly, ChIP-qPCR confirmed prominent binding of PBX1 to promoter proximal sites of the *Rfx4*, *Pbx3* and *Nfia* genes in V-SVZ neural stem- and progenitor cells (Fig. 6h). Modeling our gene expression data with the ISMARA tool hence uncovered new regulatory circuits that are downstream of PAX6 and PBX1 and likely involved in V-SVZ neurogenesis.

## Discussion

Here, we explored the possible cooperation of the neurogenic transcription factor PAX6 and the TALE-HD protein PBX1. Chromatin-immunoprecipitation in combination with depletion of *Pbx1* revealed a requirement of PBX1 for PAX6 binding to selected sites in the genome in both adult mouse V-SVZ stem- and progenitor cells and murine ES cells. Expression profiling together with viral overexpression or knockdown of *Pax*6 and *Pbx*1, alone or in combination, identified several novel candidate regulators for V-SVZ neurogenesis. These findings identify a previously unknown partnership between PAX6 and TALE-HD family proteins and contribute novel insights into the transcriptional regulatory circuits that control adult V-SVZ neurogenesis.

Even though transcription factor activity in vivo is highly specific, consensus binding motifs for most transcription factors are very common in the genome. In fact, most classical transcription factors occupy only a fraction of their possible binding sites in any given cell, raising the question of how specificity may be achieved^40^. A broadly accepted concept is that transcription factors acquire selectivity by assembling into large, multimeric transcriptional complexes, whereby the varying composition of these complexes determines the precise DNA-sequence that the complex assembles with and the strength of binding^41,42^. This concept can account for two characteristics of developmental transcription factors: their ability to simultaneously regulate large numbers of downstream genes that do not necessarily share the same spatial and temporal expression patterns, and their capacity to control transcriptional programs that guide cells through successive steps of cellular differentiation. How these programs play out during adult V-SVZ stem cells neurogenesis is still incompletely understood. The results presented here implicate PBX1 as novel PAX6 cofactor, one of the transcription factors that control multiple steps of adult V-SVZ neurogenesis^4–6,25,43^.

PBX family proteins regulate development and morphogenesis of many different organs, form context-specific, multimeric complexes with other nuclear proteins, and act in various transcriptional networks^44^. An interesting concept, brought forward first in the context of skeletal muscle development and later in connection with breast cancer progression, is that PBX1 may be capable of transcriptional pioneering^45–47^. Pioneer transcription factors engage their target sites in closed chromatin, increase chromatin accessibility for other proteins, and thereby initiate cell fate changes or cellular reprogramming^48^. Although it is still debated whether PBX1 fulfills all these criteria, it is remarkable that a pioneering function has also been proposed for PAX6^28,49^. PAX6 recruits BRG1, a component of the BAF chromatin remodeling complex, to reorganize the chromatin structure at neuron-specific genes and potentiate expression of a neurogenic program^28^. This finding shows an interesting parallel to PBX1. PBX1 binds to regulatory regions of neuronal genes in V-SVZ progenitor cells where it, among others, serves as a docking site for MEIS2 and PARP1, which then triggers removal of the linker histone H1 from the chromatin fiber and facilitates stable expression of downstream genes^18^. Thus, an intriguing albeit still somewhat speculative model is that PAX6-PBX1 heterodimers mark distinct sites in the genome for the subsequent recruitment of different types of epigenetic regulators or chromatin remodelers.

Additional transcription factors may also participate. Among them is DLX2, a homeodomain protein that plays crucial roles in neuronal differentiation and the specification of a GABAergic/dopaminergic neuronal cell fate in the adult OB^6^. DLX2 requires interaction with PAX6, as *Pax6* deletion blocks *Dlx2*-mediated neuronal specification^6^. MEIS2, on the other hand, forms heteromeric complexes with PAX6 and DLX2, suggesting that several homeodomain-containing proteins may accumulate together at the chromatin during neuronal differentiation^6,8^. Whether such clusters form sequentially or simultaneously remains to be examined. Cooperation between PAX6 and PBX1 appears to not be limited to the adult V-SVZ neurogenic system, as depletion of *Pbx1* expression also impaired PAX6 binding to the DCX promoter/enhancer in mESCs. Other physiological contexts in which PBX1 and PAX6 may functionally interact are the developing cortex and pancreas. *Pbx1* and *Pax6* are co-expressed in the embryonic forebrain, and similar cortical patterning defects are associated with the mutation of either gene^50^. Likewise, both genes show extensive co-expression in the developing pancreas, and genetic inactivation of each impairs pancreatic development with overlapping phenotypes^51,52^.

Developmental transcription factors often function as components of self-regulating, interdependent transcriptional networks in which the expression of each transcription factor is maintained and balanced by the reciprocal regulation of other transcription factors in the network^53^. A good example for this is PAX6 itself, as it drives a cross-regulatory network of transcription factors to establish neurogenic fate and initiate neuronal differentiation in the adult forebrain^28^. We here conducted an in silico regulatory motif search using the online resource ISMARA to predict putative novel, PAX6-PBX1 co-regulated transcriptional regulators that may have a role in adult V-SVZ neurogenesis. This approach identified four prominent motifs associated with the RFX3/1/4, RFX2/7, PBX1/3, and NFIA transcription factors, respectively. Indeed, members of each group are expressed in the V-SVZ neurogenic niche, and PBX1 binding to regulatory regions of these genes was experimentally confirmed by ChIP-qPCR. We therefore conclude that transcription factors associated with these motifs likely represent novel modulators of adult V-SVZ neurogenesis that are co-regulated by PAX6 and PBX1. Computational modeling of these motifs and their predicted target genes uncovered previously unknown relationships among a set of transcription factors that included both, genes with known functions in adult V-SVZ neurogenesis and novel ones. Although experimental tests of these predictions, for example by viral misexpression experiments or with the help of mouse models, are still missing, our results highlight the power of bioinformatic modeling of genome-wide data for the identification of new genes and pathways.

In conclusion, the results presented here identify PBX1 as PAX6-cofactor and argue that the PAX6-PBX1 dyad is a key component of an interdependent transcriptional network that directs adult born neural progenitor cells towards the acquisition of a GABAergic, OB neuronal phenotype.

## Material and methods

### Neurosphere assay, cell culture

Generation, propagation, and viral transduction or transfection of aNS was performed as described in^18^. Briefly, sphere-forming cells were isolated from the lateral walls of the lateral ventricle of 9–12 week old C57BL/6 mice, males and females, and propagated under non-adherent conditions in DMEM/F-12 containing 3.5 mM glucose (Gibco, Thermo Fisher Scientific), B-27 supplement (Gibco, Thermo Fisher Scientific), 20 ng/ml fibroblast growth factor-2 (FGF2, human recombinant; PeproTech) and 20 ng/ml epidermal growth factor (EGF, human recombinant; PeproTech), L-Glutamine, and penicillin/streptomycin. Generally, freshly isolated aNS, cultured for no more than 7 days (corresponding to cells no older than early passage 2) in the presence of EGF/FGF2, were used. To obtain passage 1 aNS, primary spheres were dissociated after five days in culture by treatment with Accutase (Sigma Aldrich) for 15 min at 37 °C. Differentiation was either induced by plating dissociated, single cells at a density of 1–2 × 10^5^ cells per cm^2^ on laminin-coated tissue culture dishes (ChIP or Affymerix analysis) or by plating the equivalent of 7–8 × 10^4^ cells per cm^2^ as spheres on laminin-coated coverslips (immunofluorescent staining). Differentiation was carried out in medium lacking EGF/FGF2 but supplemented with 20 ng/ml brain-derived neurotrophic factor (BDNF; PeproTech). All experiments involving animals were approved by the animal care committee of University Hospital, Goethe University, Frankfurt and the government of Hesse, are in accordance with German and EU regulations, and comply with the ARRIVE guidelines.

Mouse embryonic stem cells (line E14Tg2a.IV^29^) were grown in DMEM with high glucose (Gibco, Thermo Fischer Scientific) supplemented with 15% FCS (Biochrom), 0.01 µg/ml LIF (ProSpec), 1 mM Na-Pyruvate, 100 nM β-mercaptoethanol, L-Glutamine, penicillin/streptomycin, and MEM nonessential amino acid supplement (Thermo Fisher Scientific) without feeder layer. Neuro2a cells were grown in DMEM (Gibco), 10% SeraPlus (PAN-Biotech) and transfected with Metafectene (Biontex).

### Retroviral transduction and siRNA transfection

For retro- and lentiviral transduction, aNS were dissociated in Accutase (Sigma Aldrich), 5 million cells per sample were incubated in a fresh 10 cm tissue cell culture dish in approximately 6 ml aNS culture medium, containing EGF/FGF2 but without penicillin/streptomycin, and incubated for at least 5 h at 37 °C in the presence of the viral stocks at 4–8 × 10^5^ CFU/ml. Transduced cells were pelleted by centrifugation, washed twice in culture medium containing EGF and FGF2, and grown for additional 48 h as free-floating spheres in the presence of growth factors prior to differentiation or fixation for ChIP. Production of viral particles followed standard procedures and is detailed in Supplementary Information.

For siRNA-mediated knockdown of *Pbx1*, first passage aNS were transfected with Silencer Select siRNAs (5`-guuggaccaacgugcaau-3; 50 pmol transfected per 2 × 10^6^ cells; Thermo Fisher Scientific) or negative control siRNAs No1 (Thermo Fisher Scientific). RNA duplexes were transfected with Metafectene Pro (Biontex). When used for ChIP, aNS cells were grown for 48 h as free-floating spheres following siRNA transfection. When used for Affymetrix gene expression arrays, cells were transduced with *Pax6*-expressing retroviruses four hours after siRNA transfection, allowed to grow as free-floating spheres for additional 48 h before differentiation was induced by growth factor withdrawal and plating on laminin-coated cell culture dishes. Knockdown with shRNA-expressing lentiviruses was performed with pGIPZ lentiviral vectors carrying shRNAs directed against *Pbx1* (Dharmacon; clone ID #389310). Knockdown efficiency of the *Pbx1-*siRNAs or *Pbx1-*silencing viruses was validated by transfection of siRNAs into aNS or transfection of shRNA-containing vectors into Neuro2a cells using Metafectene Pro (Biontex) followed by qPCR or Western Blot, quantified by densitometric analysis in Image J.

### Immunohistochemical analyses

Immunohistochemical staining was performed following standard procedures with the antibodies listed in Supplementary Information. Chromogene staining was performed with a VENTANA DISCOVERY XT automated staining system, with antigen retrieval protocol Conditioner #1, Omni-Map HRP detection, counterstaining for hematoxylin.

### cDNA synthesis and quantitative real-time PCR

RNA was isolated with help of RNeasy Mini Kit (Qiagen), including on-column DNAse I digestion, reverse transcribed with the RevertAid First strand cDNA synthesis Kit (Thermo Scientific), followed by qPCR with the Absolute QPCR SYBR Green Fluorescein Mix (Thermo Scientific) on a Bio-Rad CFX Touch Real-Time PCR detection system. Primers used for amplification are listed in Supplementary Information. Gene expression was normalized to ß-actin by using the 2^−ΔΔCT^ method. Experiments were conducted at least in triplicates and plotted as S.E.M.. Statistical significance was determined by unpaired student's t-test.

### Chromatin-immunoprecipitation (ChIP)

ChIP-qPCR was performed on chromatin isolated from 1 × 10^7^ aNS cells, 1 × 10^7^ES cells, or 10 h differentiated cells derived from 1 × 10^7^ aNS cells per sample following the procedure described in^18^ and Supplementary Information. Chromatin was sheared to an average length of 200–600 bp with a Bioruptor Plus (Diagenode) and cycle numbers optimized for each cell population. Antibodies were used in concentrations given in Supplementary Information. Identical amounts of mouse IgGs served as control. Quantitative PCR assessment was carried out as described above. Experiments were conducted at least in triplicates and plotted as S.E.M.. Enrichment of the precipitated DNA was determined relative to the input (1:100) as *100* × *2*^(*Ct adjusted Input* — *Ct* IP)^. Standard error was calculated between experimental replicates. Statistical significance was assessed by unpaired student's t-test, comparison between three or more groups was carried out by one-way ANOVA followed by Bonferroni Multiple Comparison post-hoc test. Statistical significance was assumed when *p < 0.05, **p < 0.01, ***p < 0.001.

### Affymetrix gene expression arrays and data analysis

Total RNA was Biotin-labeled with the GeneChip WT PLUS Reagent Kit (Affymetrix). Hybridization, post-hybridization, and scanning were performed according to standard procedures and as further detailed in Supplementary Information. CEL files were imported into Partek Genomics Suite version 7 (Partek Inc.). Inter-individual sample variability and outliers within the datasets were assessed by principal component analysis (PCA). Differentially expressed genes (DEGs) between control and different treatment groups were identified by ANOVA analysis with genetic manipulations represented as linear contrasts. Differentially expressed genes (FDR ≤ 0.05 and fold change ≥ 1.5) were exported, displayed as a heatmap (only FDR) and analyzed for Gene Ontology enrichment using the online resource DAVID. GO terms together with corresponding p-values were exported from DAVID and further summarized as enriched biological processes using the Revigo online tool. The array data were submitted to NCBI Gene Expression Omnibus under the accession number GSE172449.

For ISMARA analysis, CEL files were uploaded and normalized within ISMARA standard mode settings including the averaging across sample replicates. Results are displayed as activity profiles showing inferred activities of regulatory motifs across samples together with activity-expression correlation values. Regulatory motifs were ranked and displayed as Z-values showing highly deregulated activities as inferred from transcriptome changes.

### Institutional review board statement

All experiments involving animals were approved by the local animal care committee and the government of Hessen, are in accordance with German and EU regulations, and comply with the ARRIVE guidelines.

### Informed consent statement

Not applicable.

## Supplementary Information

Supplementary Figures.

Supplementary Table 1.

Supplementary Table 2.

Supplementary Table 3.

Supplementary Table 4.

Supplementary Table 5.

Supplementary Table 6.

Supplementary Table 7.

Supplementary Table 8.

## Data Availability

The array data have been submitted to NCBI Gene Expression Omnibus (GEO) under the accession number GSE172449.
