# Supplementary material for: Transcriptional cooperation of PBX1 and PAX6 in adult neural progenitor cells
Source: Sci Rep. 2021 Oct 25;11:21013. doi: 10.1038/s41598-021-99968-5 (PMC8545929; doi:10.1038/s41598-021-99968-5)
Supplement: Supplementary file 1 — Supplementary Figures. [file 41598_2021_99968_MOESM1_ESM.pdf]

Transcriptional cooperation of PBX1 and PAX6 in adult neural progenitor cells

Ann-Christin Hau, Elise Mommaerts, Vera Laub, Tamara Müller, Gunnar Dittmar, Dorothea Schulte

Supplementary Information

1. Extended Material and Methods
2. Supplementary figures
3. Supplementary tables

Extended Material and Methods

Cultivation of primary stem- and progenitor cells of the SVZ as free-floating neurospheres (aNS), transduction, transfection, and differentiation

Cells were isolated from the lateral walls of the lateral ventricle of 9 to 12 week-old C57BL/6 mice, males and females, and propagated under non-adherent conditions in DMEM/F-12 containing 3.5 mM glucose (Gibco, Thermo Fisher Scientific), B-27 supplement (Gibco, Thermo Fisher Scientific), 20 ng/ml fibroblast growth factor-2 (FGF2, human recombinant; PeproTech) and 20 ng/ml epidermal growth factor (EGF, human recombinant; PeproTech), L-Glutamine, and penicillin / streptomycin. Generally, freshly isolated primary aNS, cultured for no more than 5 days in the presence of EGF/FGF2, were used. To obtain passage 1 aNS, primary spheres were dissociated after five days in culture by treatment with Accutase (Sigma Aldrich) for 15 min at 37°C.

For immunohistochemical analyses, cellular differentiation was induced as follows: First-passage aNS were dissociated and cultured in EGF/FGF2-containing medium for another 48 hours. Cell numbers were then determined by dissociating and counting cells in a small aliquot of each aNS culture, and spheres were plated at a density corresponding to 7-8x10^4 cells per cm^2 in medium without EGF/FGF2 but supplemented with 20 ng/ml brain-derived neurotrophic factor (BDNF; PeproTech) on laminin-coated tissue coverslips for another 24 hours. For ChIP or Affymetrix analysis, differentiation was induced by plating dissociated, single cells at a density of 1-2x10^5 cells per cm^2 in medium without EGF/FGF2 but supplemented with 20 ng/ml BDNF on laminin-coated tissue culture dishes as described in Hau et al., 2017. For subsequent Affymetrix analysis, cells were harvested after 10 hours of differentiation, for chromatin immunoprecipitation at the times indicated.

For retro- and lentiviral transduction, aNS were dissociated in Accutase (Sigma Aldrich), 5 million cells per sample were incubated in a fresh 10cm tissue cell culture dish in approximately 6ml aNS culture

medium, containing EGF/FGF2 but without penicillin/streptomycin, and incubated for at least 5 hours at 37°C in the presence of the viral stocks at 4-8x10^5 CFU / ml (see below). Transduced cells were pelleted by centrifugation, washed twice in culture medium containing EGF and FGF2, and grown for additional 48 hours as free-floating spheres in the presence of growth factors prior to fixation for ChIP or differentiation.

For siRNA-mediated knockdown of *Pbx1*, first passage aNS were transfected with Silencer® Select siRNAs (5`-guuggaccaacgugcaau-3; 50pmol transfected per 2x10^6 cells; Thermo Fisher Scientific) or negative control siRNAs No1 (Thermo Fisher Scientific). RNA duplexes were transfected with Metafectene Pro (Biontex). When used for ChIP, aNS cells were grown for 48 hours as free-floating spheres following siRNA transfection. When used for Affymetrix gene expression arrays, cells were transduced with Pax6-expressing retroviruses as described above four hours after siRNA transfection, allowed to grow as free-floating spheres for additional 48 hours before differentiation was induced by growth factor withdrawal and plating on laminin-coated cell culture dishes. Knockdown with shRNA-expressing lentiviruses was performed with pGIPZ lentiviral vectors carrying shRNAs directed against *Pbx1* (Dharmacon; clone ID numbers are given in Figs. S2). For the Pbx1KD experiments shown in Figs. 3-6, pGIPZ-Pbx1sh clone #389310 was used. Lentiviral transduction was performed as described for retroviral transduction. Knockdown efficiency of the *Pbx1*-siRNAs or *Pbx1*-silencing viruses was validated by transfection of siRNAs into aNS or transfection of shRNA-containing vectors into Neuro2a cells using Metafectene Pro (Biontex) followed by qPCR or Western Blot, quantified by densitometric analysis in Image J, Version 1.5.1, rel. April 23, 2018 (https://imagej.nih.gov/ij/all-notes.html) (Fig. S2).

Cell culture

Mouse embryonic stem cells (line E14Tg2a.IV) were grown in DMEM with high glucose (Gibco, Thermo Fischer Scientific) supplemented with 15% FCS (Biochrom), 0.01µg/ml LIF (ProSpec), 1mM Na-Pyruvate, 100nM β-mercaptoethanol, L-Glutamine, penicillin / streptomycin, and MEM nonessential amino acid supplement (Thermo Fisher Scientific) without feeder layer. For *Pbx1*-knockdown, ES cells were transduced with pGIPZ-Pbx1 shRNA clone #389310 following the same protocol as detailed above for aNS lentiviral transduction. Neuro2a cells were grown in DMEM (Gibco, Thermo Fisher Scientific), 10% SeraPlus (PAN-Biotech) and transfected with Metafectene (Biontex).

Production of retro- or lentiviral particles.

Retroviral transduction was carried out with pCLIG, a GFP-expressing vector virus [1]. pCLIG-Pax6 contains the coding sequence of canonical Pax6, pCLIG-Pbx1 contains the coding region of Pbx1b. Retroviral particles were generated in HEK293T cells with the packaging plasmids pUMVC (Addgene #8449) and pCMV-VSV-G (Addgene #8454). Lentiviral particles were generated in Hek293T cells using a 2nd generation packaging system.

Immunohistochemical assays: Immunostaining, FACS, Western Blot

For immunofluorescence analysis of *in vitro* grown aNS or neurons, the cells were fixed with 4% paraformaldehyde (PFA) in phosphate buffered saline pH 7.5 (PBS) for 10 minutes at RT and washed in PBS at 4°C. aNS were allowed to attach to poly-D-lysine-coated coverslips for 30min at 37°C prior to fixation. Primary and secondary antibodies were diluted in 10% goat serum and 0.5% TritonX-100 in PBS. Primary antibodies were applied over night at 4°C. Samples were washed three times with PBS for 5-10 minutes each. Secondary antibodies were applied for one hour at RT. The samples were washed with PBS, cell nuclei were stained with 4',6- diamidino-2-phenylindole (DAPI), and samples were mounted with Aqua Poly Mount (Polyscience Inc.).

Antibody; species	Distributor	Dilution for IFC
Pbx1; rb	Cell Signaling Technology, #4342	1:15,000 (custom formulated)
PAX6; ms	DHSB supernatant (Fig. 1a, d, e)	undiluted
PAX6; rb mAb	Cell Signaling Technology, D3A9V (Fig. 1j, k)	1:250
DCX; gp	Merck Millipore, #AB2253	1:5,000
GFAP; ms	Sigma Aldrich, G3893	1:2,000
NESTIN; ms	BD Pharmingen 556309, clone rat 401	1:2,000

Secondary antibodies were Alexa 488-, Alexa 568-, or Alexa 647-conjugated (Molecular Probes, Thermo Fisher Scientific). Marker combinations analyzed were NESTIN/DCX/PBX1, NESTIN/DCX/PAX6, DCX/GFAP/PBX1, DCX/GFAP/PAX6. Images were taken with a Nikon 80i, a Nikon Eclipse TE2000-E or a Nikon Ti2 confocal microscope with optical sections of 0.5-2 µm intervals for confocal images. Quantification was carried out in the NIS Elements software. For chromogen staining, adult C57bl6 mice were sacrificed by cervical dislocation, the brains were immersed in Tissue-Tek O.C.T. (Sakura Finetek) without further fixation and kept overnight at -20°C. Cryostat sections were cut on a Microm HM550 with a feather microtome blade at a thickness of 15µm. Postfixation was 10min at RT in 2% PFA. Antigen detection was performed with a VENTANA DISCOVERY XT automated staining system, with antigen retrieval protocol Conditioner #1, Omni-Map HRP detection and counterstaining for hematoxylin.

Fluorescent cell sorting (FACS) was performed with anti-PSA-NCAM-PE, #130-117-394, IgM-PE, #130-120-156 (both MACS Miltenyi Biotec), or anti-α6 integrin (R&D Systems, FAB 13501A) on a Becton Dickinson FACS Canto.

Western Blot was carried out following standard procedures and with these antibodies: β-actin (abcam ab8227, 1:2,500), Lamin B (abcam ab16048, 1:4,500), PAX6 (Covance PRB-278P, 1:5,000), PBX1 (Cell Signaling Technology #4342 (custom formulated), 1:50,000).

RNA Isolation and cDNA transcription and quantitative real-time PCR

aNS or *in vitro* differentiated neurons were collected in ice-cold PBS and RNA was isolated with the RNeasy Mini Kit (Qiagen), including on-column digestion of remaining genomic DNA. mRNA was reverse transcribed with the RevertAid First strand cDNA synthesis Kit (Thermo Fisher Scientific), followed by qPCR with the Absolute QPCR SYBR Green Fluorescein Mix (Thermo Fisher Scientific) on a BioRad CFX Touch Real-Time PCR detection system. Primers used for the amplification are listed below. Gene expression was normalized to ß-actin by using the $2^{-\Delta\Delta CT}$ method. Each experimental value thereby represents three independent measurements. Experiments were conducted at least in triplicates and plotted as S.E.M.. Statistical significance was determined by unpaired student's t-test. Primers used were:

**Primer**	**Sequence (5`-3`) fwd**	**Sequence (5`-3`) rev**
Ablim3	tggagagatgcggttatggagag	agcggtagtaatgaggcgagc
Atat1	tgtctggtcccgcacaatg	ccttgcccagctcatctaa
b-actin	agccatgtacgtagccatcc	ctctcagctgtggtggtgaa
Dcx	ggaaggggaaagctatgtctg	ttgctgctagccaaggactg
Meis1	tggaattagagaaggtacacgaa	gttcctcctgaacgagtgga
Meis2	aggtgatgacgacgatccag	ggcattgataaaccagttgttcac
Pax6	atcataactccgcccattca	tacctgaagcaagaatacagg
Pbx1	atcatccaccgcaagttcag	gtttgatacctgggagactgtg

Chromatin-immunoprecipitation (ChIP)

ChIP was performed as described [2]. $1x10^7$ aNS cells, ES cells, or 10h differentiated cells derived from $1x10^7$ aNS cells were used for each ChIP. Chromatin was cross-linked in PFA pH7.4, 2% when freshly prepared from powder or 1% when diluted from a 16% stock (Electron Microscopy Sciences). Cross-

linking conditions were 23 minutes at 4°C rotating for aNS, or 10 minutes at RT for *in vitro* differentiated neurons, glia, or ES cells. Chromatin was sheared to an average length of 200-600bp with a Bioruptor Plus (Diagenode) with cycle numbers optimized for each cell population. Antibodies were used in concentrations indicated in the table below, identical amounts of mouse IgGs served as control. Quantitative PCR assessment was carried out with Absolute QPCR SYBR Green Fluorescein Mix (Thermo Fisher Scientific) and a Bio-Rad CFX Touch or Bio-Rad MyiQ Real-Time PCR detection system. Experiments were conducted at least in triplicates and plotted as S.E.M.. Enrichment of the precipitated DNA was determined relative to the input (1:100) as $\mathbf{100 \times 2^{(Ct\ adjusted\ Input\ -\ Ct\ IP)}}$. Standard error was calculated between experimental replicates. Statistical significance was assessed by unpaired student's t-test, comparison between three or more groups was carried out by one-way ANOVA followed by Bonferroni Multiple Comparison post-hoc test. Statistical significance was assumed when * $p<0.05$, ** $p<0.01$, *** $p<0.001$.

**Antibody**	**Distributor**	**Concentration per ChIP**
Pbx1	Cell Signaling Technology #4342	1:115 (standard formulated; 2µg)
H3K4me³	Cell Signaling Technology #9751 clone C24D8	1:545 (2µg)
H3K27me³	Cell Signaling Technology #9756	1:268 (2µg)
Histon H1.4	Millipore 05-457	1:450 (1µg)
mouse IgG	Merck Millipore/ Upstate #12-371B	1:180 – 1:545 (2µg)
Meis1/2, N-17	Santa Cruz sc-10600X	1:180 (2µg)
acCBP/p300	Cell Signaling Technology #4771	1:450 (2µg)
RNApol II	Millipore #05-623	1:450 (2µg)

**Primer**	**Sequence (5`-3`) fwd**	**Sequence (5`-3`) rev**
Dcx-2.728	gcatatctgtgtttatggctgc	ctcggatacttcactcagtatatc
Dcx -1.957	aaacctttctagctgttaatgcagg	ctccaagcaagaaattcctgccagggtg
Dcx +7.624	cattagagaatcattctccattttcc	gactgtccacctttaagtattcttc

Dcx primers out (-12.597)	gtttctgatagacaagtatacttagg	cctataacaagggttagatctcagc
Sox11 pos.-4	ctgaggtggtcagtaaaagtctct	atcctgaggacatcctggct
Sox11 pos.-7	actatagaactgtccaacacatggg	cacaccatactcttgtttctgagtt
Myogenin (Mgn)	caacccaggagatcatttgct	acagacatatcctccaccgt
Rfx4	ttcggaggcggcttttcttat	gaatggccgctggtcagatg
Pbx3	tccctcaaggagtgaccagt	agtgtgcaaagcgcagttag
Nfia	cctgctgcaatgtcgagaaa	aagctgccaagtgtgtagga

Gene expression analysis

RNA was isolated with the RNeasy Mini Kit (QIAGEN) including on-column DNaseI digestion, followed by EtOH precipitation to ensure sufficient RNA quality. RNA concentration and quality were assessed using the Fragment Analyzer (Advanced Analytical Technologies Inc.). Briefly, 40ng of total RNAs were transcribed in cDNA and labelled with Biotin using the GeneChip WT PLUS Reagent Kit (Affymetrix) according to the manufacturer's standard protocol (P/N 703174 Rev. 2). Labelled cDNA products were randomly fragmented and hybridized with 5.5µg onto each Mouse Gene 1.0 ST arrays (Affymetrix) using the GeneChip Hybridization Oven 645 (Affymetrix). After hybridization, arrays were washed and stained using the Affymetrix GeneChip WT Terminal Labeling and Hybridization Kit together with the GeneChip Fluidics Station 450 (Affymetrix), before being scanned using a GeneChip Scanner 3000 7G (Affymetrix). After scanning, CEL files were imported into Partek Genomics Suite version 7 (Partek Inc.) and collectively normalized using the Robust Multi-Array (RMA) method. Principal component analysis was used to identify inter-individual sample variability and outliers within the datasets. Analysis of variance (ANOVA) with genetic manipulations represented as linear contrasts was used to detect differentially expressed genes (DEGs) between control and different treatment groups. Differentially expressed genes (FDR $\leq$0.05 and fold change $\geq$1.5) were exported, displayed as a heatmap (only FDR) and analysed for Gene Ontology enrichment using the online resource DAVID (https://david.ncifcrf.gov/) [3]. GO terms together with corresponding p-values were exported from DAVID and further summarised as enriched biological processes using the Revigo online tool (http://revigo.irb.hr/) [4].

Draw Venn Diagramm (http://bioinformatics.psb.ugent.be/webtools/Venn/) was used to create intersecting lists of DEGs for different comparisons. The array data were submitted to NCBI Gene Expression Omnibus under the accession number GSE172449.

ISMARA analysis

ISMARA was used to potentially identify key transcription factors which might be involved in driving the gene expression changes observed in the transcriptome expression data from Affymetrix GeneChip arrays [5]. To this end, CEL files were uploaded and normalized within ISMARA standard mode settings including the averaging across sample replicates. Results are displayed as activity profiles showing inferred activities of regulatory motifs across samples together with activity-expression correlation values. Furthermore, regulatory motifs were ranked and displayed as Z-values showing highly deregulated activities as inferred from transcriptome changes.

References

1. Hojo, M. *et al.* Glial cell fate specification modulated by the bHLH gene Hes5 in mouse retina. *Development (Cambridge, England)* **127,** 2515–2522 (2000).
2. Hau, A.-C. *et al.* MEIS homeodomain proteins facilitate PARP1/ARTD1-mediated eviction of histone H1. *The Journal of cell biology* **216,** 2715–2729 (2017).
3. Huang, D. W., Sherman, B. T. & Lempicki, R. A. Systematic and integrative analysis of large gene lists using DAVID bioinformatics resources. *Nature protocols* **4,** 44–57 (2009).
4. Supek, F., Bošnjak, M., Škunca, N. & Šmuc, T. REVIGO summarizes and visualizes long lists of gene ontology terms. *PloS one* **6,** e21800 (2011).
5. Balwierz, P. J. *et al.* ISMARA: automated modeling of genomic signals as a democracy of regulatory motifs. *Genome research* **24,** 869–884 (2014).

Supplementary Figures

Figure S1

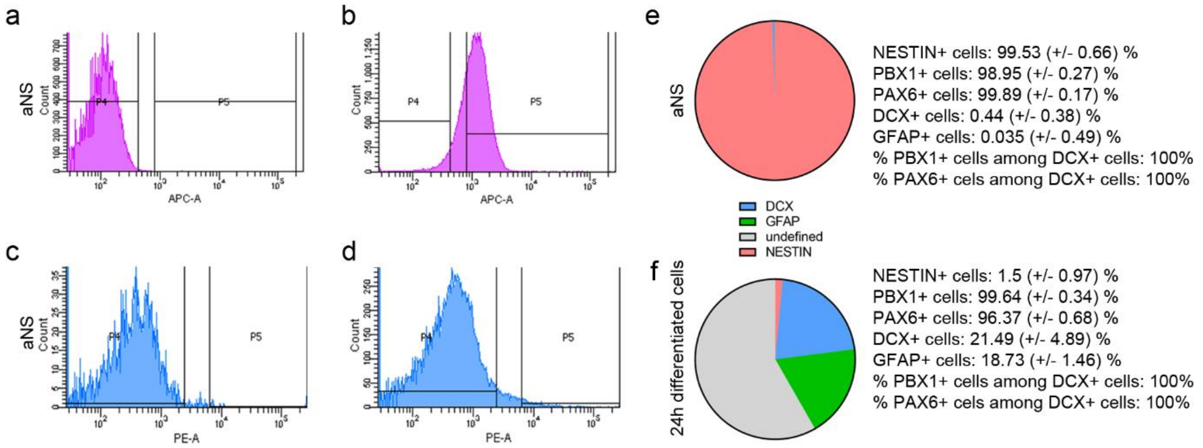

Figure S1: Cellular composition of V-SVZ-derived aNS and 24h differentiated cell cultures. (a-d) Sphere-forming cells isolated from the V-SVZ were analyzed by FACS at day 3, passage 1 (i.e. after three days of cultivation under proliferative conditions following the first passaging). (a, b) Sorting for integrin α6-positive stem- and progenitor cells: (a) unstained control with 0.0% in P5 (background for integrin α6-staining); (b) integrin α6-staining with 74.1% positive cells in P5. (c, d) Sorting for PSA-NCAM-positive neuroblasts: (c) IgM-PE staining with 0.5% cells in P5 (background for PSA-NCAM staining with isotype control). (d) PSA-NCAM-PE staining with 1.3% positive cells in P5. *n*=2. (e) Quantification of fluorescent immunostainings for NESTIN, DCX and GFAP (counterstained for DAPI) in sphere-forming aNS; pie chart for NESTIN+, DCX+ or GFAP+ cells (left), quantification of PBX1+ and PAX6+ cells (right; *n*=3-5; on average 2,150 cells counted each). (f) Pie chart and quantification of NESTIN+, DCX+ or GFAP+ cells in 24 hours-differentiated cells and proportion of PBX1+ and PAX6+ cells among them (*n*=3; on average 1,190 cells counted each). Undefined: cells negative for DCX, GFAP or NESTIN. Oligodendrocytes were not assessed for their protracted maturation.

Figure S2

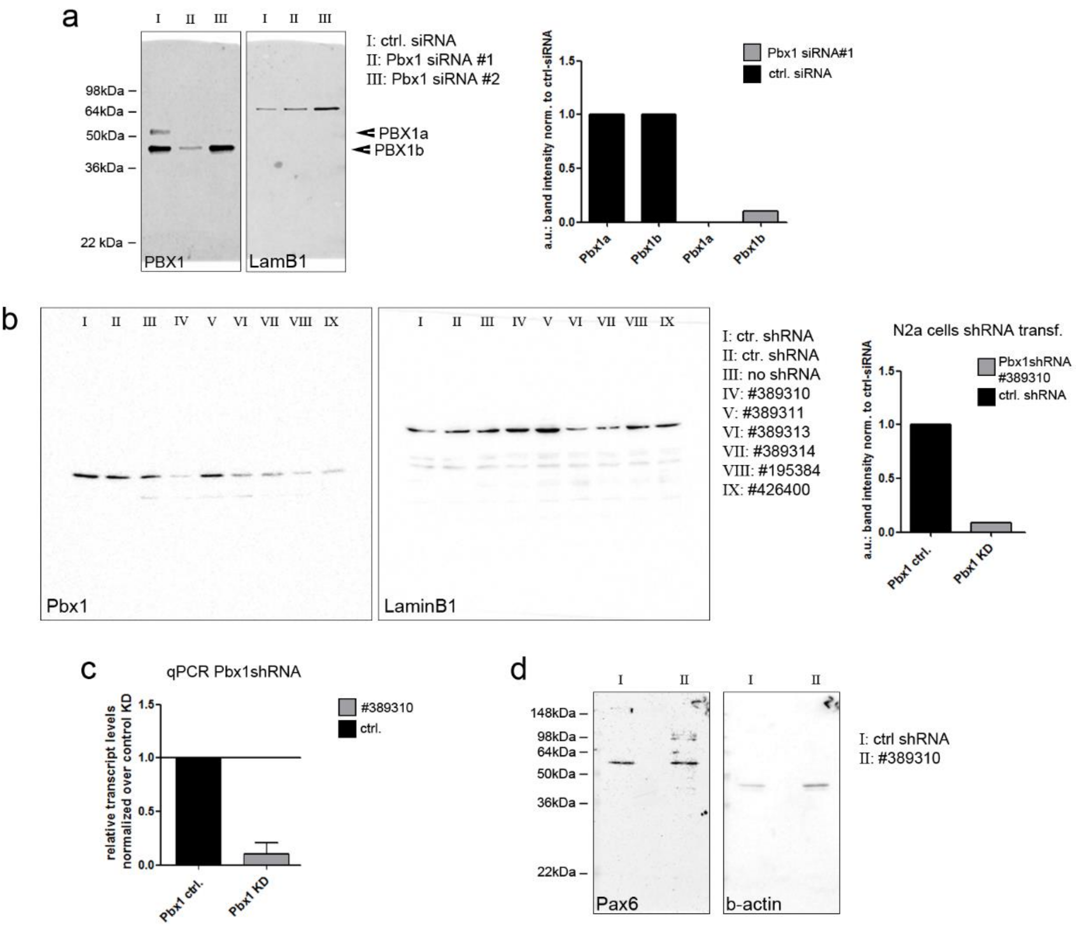

Figure S2: Validation of siRNA and shRNA KD strategies. (a) Western Blot of nuclear extracts prepared from aNS following transfection of siRNAs directed against *Pbx1* or non-targeting control siRNAs (left) and densitometric quantification of the results (right). The same blot was first analyzed for PBX1, then re-probed for LamB1 as loading control; band intensities for PBX1 were normalized to LamB1 and are expressed as ctrl siRNA = 1. Pbx1 siRNA #1, showing robust depletion of isoforms PBX1a and PBX1b was used for further experiments. (b) Western Blot of nuclear extracts prepared from Neuro2a cells following transfection with the shRNA vector viruses as indicated. shRNAs are identified by catalog No. shRNA #389310 was used for all further experiments (left); densitometric quantification of the KD achieved with shRNA #389310 (right). Band intensities for PBX1 were normalized to LamB1 and are expressed as ctrl shRNA = 1. (c) qPCR validation of the KD achieved with shRNA #389310 in aNS. (d) Western Blots for PAX6 and β-actin on aNS extracts treated with ctrl shRNAs (Thermo Fischer Scientific) or shRNA #389310. *Pbx1K*D does not affect PAX6 protein abundance, excluding the possibility that the observed reduced PAX6 binding to genomic loci is an indirect consequence of reduced PAX6 availability. Densitometric quantification of protein band intensities on Western Blots was performed

in Image J, Version 1.5.1., April 23 2018 (https://imagej.nih.gov/ij/all-notes.html). For original membranes to (a, b, d), please see Figure S5.

Figure S3

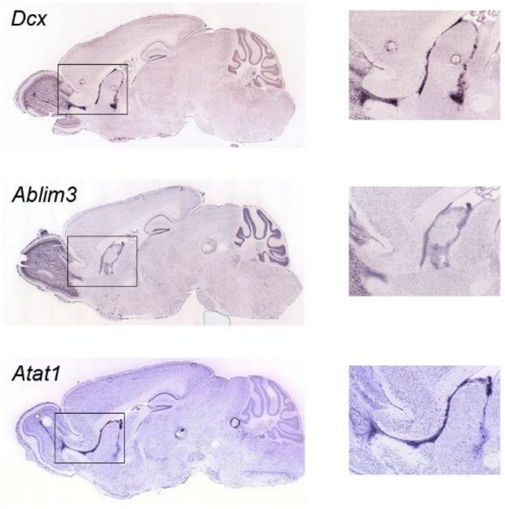

Figure S3: Expression of PAX6-PBX1 co-regulated genes *Dcx*, *Ablim3* and *Atat1* in the adult mouse brain. *In situ* hybridization for the genes indicated on sagital sections of adult mouse brains. Higher magnifications of the boxed areas are shown on the right. Note the prominent expression of *Ablim3* and *Ata1* in the V-SVZ and RMS, two regions where adult generated progenitor cells and neuroblasts, respectively, are present. *Dcx*, a known marker for migrating neuroblasts in the V-SVZ, RMS and olfactory bulb, is shown for comparison. Images are taken from the Allen Mouse Brain Atlas (https://mouse.brain-map.org/).

Figure S4

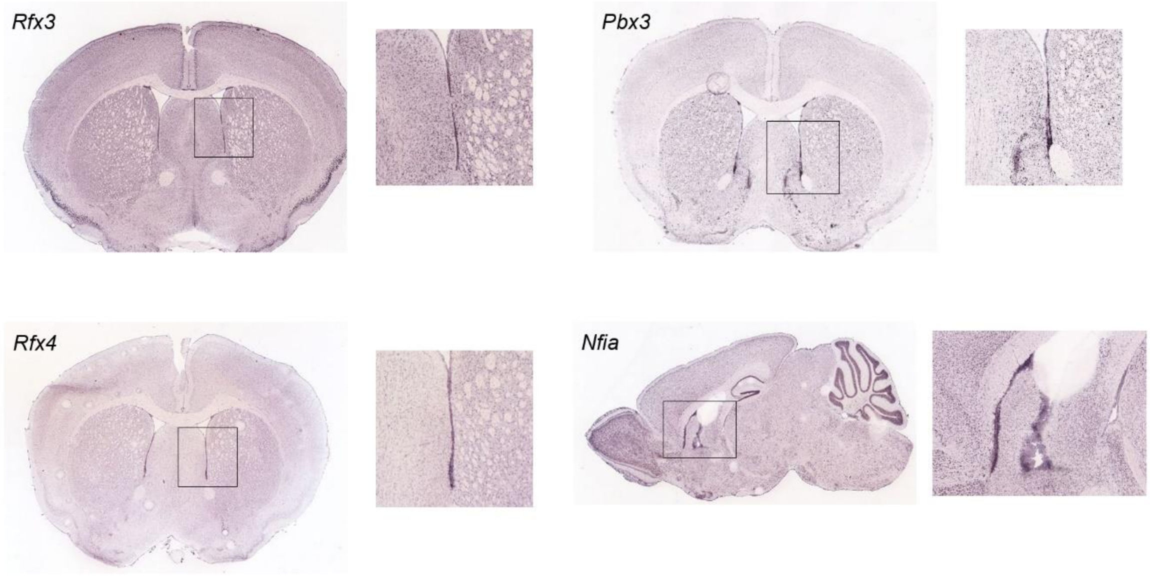

Figure S4: Expression of TFs predicted by ISMARA-modeling to be likely relevant for the observed gene expression differences between the *Pax6*OE, *Pax6*OE/*Pbx1*KD and vector ctrl datasets. *In situ* hybridization for four TFs, which are known to bind to motifs whose activity was predicted by ISMARA

to account for gene expression differences between the *Pax6*OE, *Pax6*OE/*Pbx1*KD and vector ctrl samples. Coronal sections through the forebrain of adult mice are shown for Rfx3, Rfx4 and Pbx3, a sagital section for Nfia. The boxed areas indicating the V-SVZ in the coronal sections or the transition from V-SVZ to RMS in sagital section are shown at higher magnifications on the right hand side. Note the prominent expression of all four TFs in the V-SVZ germinal niche. Images are taken from the Allen Mouse Brain Atlas (https://mouse.brain-map.org/).

Supplementary Tables

Table S1: Differentially expressed genes (DEGs) between normalized Pax6OE/Pbx1KD (abbreviated Pbx1KD) andPax6 OE datasets

Table S2: Differentially expressed genes (DEGs) between normalized Pbx1 OE versus vector ctrl datasets

Table S3: Differentially expressed genes (DEGs) between normalized Pax6OE versus vector ctrl datasets

Table S4: Functional annotation chart from the DAVID online resource

Table S5: Collective list of DEGs from both comparisons i) normalized Pax6OE/Pbx1KD (abbreviated Pbx1KD) and Pax6OE datasets and ii) normalized Pax6OE versus vector ctrl datasets

Table S6: ISMARA analysis motifs sorted by significance.

Table S7: Gene overrepresentation in biological process category exported from ISMARA for the Rfx3_Rfx1_Rfx4 motifs

Table S8: Gene overrepresentation in biological process category exported from ISMARA for the Nfia motif

Figure S5

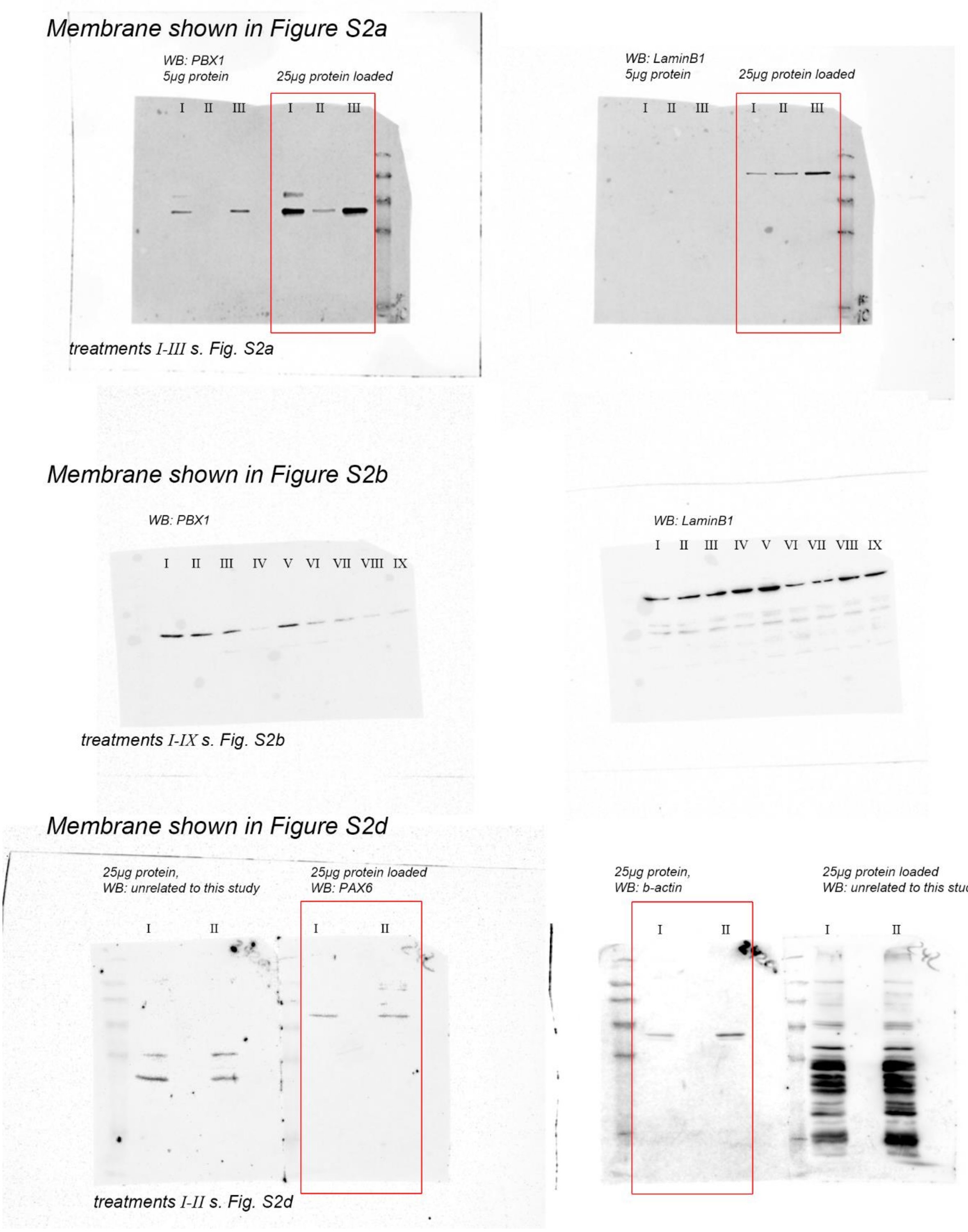

Extracts I-III were loaded at 5µg and 25µg protein per lane on the gel shown in Fig. S2a; extracts I and II were loaded twice at 25µg per lane on the gel shown in Fid. S2d. Red boxes mark the areas of the membranes shown in Fig. S2a and S2d, respectively.
